# Construction and Practice of the County Integrated Healthcare System in China: A Case Study of Shaxian, Sanming

**DOI:** 10.5334/ijic.9542

**Published:** 2026-08-18

**Authors:** Lina Zhao, Yiyang Chen, Fanyi Kong, Zhiyi Luo, Yazi He, Zongjiu Zhang

**Affiliations:** 1 School of Biomedical Engineering, Tsinghua University, Beijing, China; 2 Institute for Hospital Management, Tsinghua University, Shenzhen, China

**Keywords:** integrated care, county-level medical community, Sanming medical reform, grassroots capacity

## Abstract

**Introduction:**

Integrated healthcare is a global health reform trend to enhance population health and well-being through person-centered service integration. AS one of the world’s most populous developing countries, China has prioritized building county-level medical communities (CMCs) to tackle healthcare challenges, integrate resources, strengthen primary-level capacity, and improve hierarchical service delivery.

**Description:**

This paper examines the construction of the CMC in Shaxian District, Sanming City, Fujian Province, as a representative case study. It systematically reviews key initiatives, outcomes, institutional frameworks, management approaches, and operational mechanisms deployed in the district’s healthcare integration efforts.

**Discussion:**

The Sanming model, exemplified by the Shaxian case, demonstrates how strategic resource integration, standardized management, and mechanism innovation can improve service efficiency and equity at the grassroots level. Its success provides transferable lessons in policy design, cross-sectoral collaboration, and performance evaluation for advancing integrated healthcare.

**Conclusion:**

The Shaxian CMC provides valuable insights and actionable policy recommendations for countries and regions advancing integrated healthcare systems, offering potential for global adaptation to strengthen primary care and health equity.

## Introduction

Integrated care is a globally acknowledged paradigm in healthcare systems [1]. It encompasses two primary dimensions: integration, which aims to coordinate fragmented elements across the healthcare continuum, and service quality, which ensures timely and effective delivery of care to patients [2]. The model’s core objectives include enhancing healthcare efficiency, optimizing patient experience, and improving clinical outcomes [3]. Globally, nations have developed distinct integrated care models to address aging populations, chronic disease burdens, and system fragmentation. The United States employs the insurer-led Kaiser model [4,5]; the United Kingdom utilizes its GP system to coordinate three-tiered primary healthcare integration [6,7]; Singapore’s Agency for Integrated Care (AIC) bridges healthcare and social services [8]; while Japan implements community-based service coordination in legal frameworks [9].

AS one of the world’s most populous developing countries, China confronts unique structural healthcare challenges: regional resource disparities, underdeveloped grassroots capacity, and unsustainable cost growth [10]. Consequently, China is developing distinctive integrated care approaches to improve resource efficiency and service quality [11]. The State Council’s 2017 policy established hierarchical medical alliances [12], with county-level medical community (CMC) models integrating county hospitals, township centers, and village clinics into coordinated systems. This “Healthy China” initiative aims to (1) strengthen county-level services, (2) optimize resource allocation, and (3) standardize care delivery protocols. Table 1 details China’s CMC policy framework.

**Table 1:** Policy Review of County-level Medical Community Development in China [13].


STAGE	YEAR	POLICY TITLE	CORE CONTENT

Exploratory Preparation Stage	2015	*Guiding Opinions on Promoting the Construction of Hierarchical Medical System*	Emphasized the provision of healthcare services by different medical institutions through hierarchical classification, and integrated regional medical resources.

	2015	*Implementation Opinions on Comprehensively Promoting the Reform of County-Level Public Hospitals*	Strengthened medical planning at the county level, explored the establishment of healthcare community models, and advanced the hierarchical medical system.

	2016	*Guiding Opinions on Piloting the Construction of Medical Consortia*	Clarified four organizational models of medical consortia: urban medical consortia, county-level medical communities, specialized medical alliances, and telemedicine collaboration networks.

Pilot Development Stage	2017	*Guiding Opinions on Promoting the Construction and Development of Medical Consortia*	Launched multi-form pilot projects for medical consortia, promoted classified development according to local conditions, and further improved working mechanisms, resource support, and policy frameworks.

	2018	*Work Plan for Comprehensive Performance Evaluation of Medical Consortia*	Formulated performance evaluation schemes for medical consortia, improved relevant systems, and emphasized the feedback and timely application of evaluation results.

Construction and Improvement Stage	2019	*Notice on Promoting the Construction of Compact County-level Medical Communities*	The first policy document specifically targeting compact county-level medical communities, specifying pilot counties, detailed work requirements, and initial construction objectives.

	2020	*Opinions on Deepening the Reform of Medical Security Systems*	Explored global budget payment for compact healthcare communities, strengthened supervision and evaluation, and proposed mechanisms such as retained surpluses and reasonable overspending sharing.

	2020	*Notice on Issuing the Administrative Measures for Medical Consortia (Trial)*	Stressed the standardization of construction and management for county-level medical communities and other medical consortia, and improved operational and management mechanisms for medical consortia.

	2020	*Evaluation Criteria and Monitoring Index System for Compact County-level Medical Communities*	The first evaluation index document for compact county-level medical communities, emphasizing the need to further enhance healthcare service capabilities at the county level and regularly monitor construction progress and effectiveness.

Experience Promotion Stage	2021	*Notice on Promoting the Experience of Sanming City in Hierarchical Medical System and Medical Consortium Construction*	Summarized the main experiences of Sanming City in hierarchical medical system and medical consortium construction, and proposed key tasks for promoting hierarchical medical system and healthcare community construction nationwide.

	2021	*Notice on Implementing the Opinions on Deepening the Reform of Medical and Health Systems by Promoting the Experience of Sanming City, Fujian Province*	Established a monitoring and evaluation mechanism for deepening the promotion of Sanming medical reform experience, and scheduled regular progress updates on policy implementation.

	2023	*Opinions on Further Deepening Reform to Promote the Healthy Development of Rural Healthcare Systems*	Highlighted the importance of rural healthcare work in rural revitalization, and advocated for improving high-quality and efficient rural healthcare systems adapted to rural characteristics, with a focus on primary medical institutions.

Comprehensive Advancement Stage	2023	*Guiding Opinions on Comprehensively Promoting the Construction of Compact County-level Medical Communities*	Put forward specific tasks and requirements for comprehensively advancing the construction of compact county-level medical communities: striving to basically complete the construction in over 90% of counties nationwide by the end of 2025, and achieving full coverage by the end of 2027.


In general, county-level medical communities have exhibited notable institutional innovations and governance achievements in practice. After nearly a decade of progressive development, China’s CMC initiative has yielded numerous influential pilot models demonstrating scalable implementation. Existing studies have documented varied CMC implementations across pilot counties, highlighting context-specific adaptations and outcomes. For instance, research on Zhejiang’s Deqing County emphasizes its resource integration and information-sharing platforms [14], while Henan’s Yindu District has been recognized for its collaborative mechanism of total prepayment per capita and DRG payment [15]. Comparative analyses reveal divergent approaches in governance structure, financial integration, and primary care empowerment, underscoring the significance of local policy experimentation and institutional adaptability [16,17]. At present, diverse approaches have emerged in the reform of county-level medical communities across China, ranging from government-led models with group-level legal entities to explorations of multi-stakeholder collaboration. Evidence shows that unified management and resource integration can enhance the efficiency of countywide medical services, improve drug procurement and institutional regulation, and strengthen the capacity of primary care to some extent [18]. In terms of payment mechanisms, global budgets, capitation, and bundled payments provide economic incentives that facilitate cost control and service coordination, though their actual effects vary across regions due to differences in local conditions and system design [19]. Meanwhile, the development and integration of information platforms contribute to knowledge sharing, organizational cohesion, and reduced coordination costs [20]. Nonetheless, several challenges persist in the reform process, including difficulties in inter-institutional coordination, limited capacity of leading hospitals, weak incentives at the grassroots level, inadequate alignment of personnel and health insurance policies, and insufficient inclusion of private institutions [21].

Notably, Sanming City’s reforms in Fujian Province have gained national prominence through their comprehensive system redesign, innovative governance frameworks, and measurable enhancements in primary care delivery, establishing the region as a benchmark for county-level integrated healthcare development in China [22,23]. Building on this foundation, Shaxian District, an integral component of the Sanming reform model, provides a valuable case for analyzing the implementation and performance of CMC policies within a well-characterized regional context.

This study takes Shaxian District as a case and adopts a systematic case study approach with three objectives: (1) to review the implementation of the CMC in Shaxian, (2) to analyze the internal management measures and operational mechanisms, and (3) to summarize the lessons learned from the construction process. The research systematically organizes and analyzes policy documents, institutional materials, and key informant interviews to identify replicable policy innovations. Findings aim to provide evidence-based guidance for health system reformers in low- and middle-income countries (LMICs) pursuing integrated care models under resource constraints.

Beyond summarizing local reform experience, this study develops an analytical framework for understanding how county-level integrated care can be advanced under resource-constrained and government-led conditions. The Shaxian case shows that such integration depends not only on organizational restructuring, but also on the realignment of economic incentives across healthcare delivery, medical insurance, and pharmaceutical governance. This perspective complements the WHO’s people-centered integrated care framework [24] and the Rainbow Model of Integrated Care, which conceptualizes integration across clinical, professional, organizational, system, functional, and normative dimensions [25]. It further highlights incentive adjustment as an enabling condition for integration in low- and middle-income country contexts.

### Case Selection: Regional Overview and Healthcare Development Achievements

Shaxian District was selected because it represents a concrete county-level implementation of the Sanming reform. The Sanming experience in the construction of a hierarchical medical system and medical consortia has been promoted through national policy documents, making it an important reference point for China’s primary healthcare system reform. Shaxian also has distinctive analytical value because it combines the typical county-township-village structure of China’s CMC reform with key Sanming mechanisms, including unified governance, bundled payment, and health-oriented performance evaluation.

In this context, Shaxian District provides a suitable county-level setting for analysis. Located in central Fujian Province, it covers an area of 1,815 square kilometers [26]. According to the 2024 Statistical Bulletin of Shaxian District, the district had a permanent resident population of 246,000 and a GDP of 33.076 billion yuan in 2024. By the end of 2024, Shaxian had 259 health institutions, including hospitals, township health centers and branches, community health service institutions, private and individual medical institutions, public health institutions, and village clinics. Public medical and health institutions in the district employed 1,755 professional health technicians, including 663 licensed physicians and assistant physicians and 827 registered nurses. The district had 1,391 beds across health institutions, including beds in the district general hospital, township health centers, private institutions, and the maternal and child health hospital [27].

As early as 2012, Sanming City initiated a comprehensive public hospital reform program, which became widely known as the “Sanming medical reform”. In 2013, Sanming launched a compensation system reform, under which 22 hospitals at or above the secondary level adopted an annual salary system for hospital directors and healthcare professionals, effectively decoupling individual remuneration from departmental revenue. Additional key measures included the complete elimination of drug markups and the centralized administration of medical insurance funds. In 2017, Shaxian initiated the establishment of a county-level medical community, led by Shaxian General Hospital and supported by 12 township health centers and 128 village clinics. In 2019, Shaxian General Hospital was included on the National Health Commission’s list of county hospitals meeting the national standards for comprehensive service capacity. In March 2021, Chinese President Xi Jinping visited Shaxian General Hospital and emphasized the importance of building upon the existing foundation to further advance the healthcare sector.

With the ongoing development of the county-level medical community, Shaxian has achieved significant outcomes. In recent years, Shaxian District has established a hierarchical medical service system. In 2024, medical institutions recorded 11,000 two-way referrals [28], and primary medical institutions accounted for 58% of all outpatient visits [29], exceeding the national average. Furthermore, Sanming area has actively promoted family doctor contracting services, achieving a signing rate of 53.32% among the permanent resident population and 85.17% among key target populations [30]. In terms of health management, Shaxian has established 215,000 electronic health records, positioning itself among the leading counties in China in terms of digitalization and information-based healthcare services [31].

## Methods

### Research Design

This study adopts a single-case study approach, focusing on the construction of the CMC in Shaxian District, Sanming City. Case study methodology is suitable for exploring complex policy practices and organizational mechanisms, as it allows for the examination of the logical connections among institutional design, management practices, and service outcomes within a specific context [32]. Shaxian was selected as an information-rich single case because it is representative of China’s county-level medical community reform and closely embedded in the nationally recognized Sanming healthcare reform. Its county-township-village structure, unified governance, and payment-based incentive mechanisms make it suitable for examining how institutional arrangements and governance mechanisms interact in a concrete county-level setting.

### Data Sources

The study draws on three main types of data:

1. Policy and official documents: including health reform policy documents, consortium construction plans, annual work reports, and publicly available statistical data issued by the Sanming and Shaxian health authorities.
2. Institutional records: including annual patient statistics, two-way referral data, and family doctor contract information provided by Shaxian General Hospital and its affiliated township health centers and village clinics.
3. Expert interviews: primary data collected through semi-structured interviews. A total of eight key informants were interviewed, including six managers from the Sanming municipal government, health authorities, Shaxian General Hospital, and township health centers, as well as one physician from a township hospital and one from a village clinic. The interviews focused on accountability, organizational management, service orientation, and benefit distribution within the consortium, resulting in approximately 25 pages of transcripts.

### Analytical Procedures

The research team systematically organized and analyzed the collected materials through an iterative thematic analysis process.

**(1) Initial coding:** Policy documents, institutional records, and interview transcripts were reviewed repeatedly. Relevant information was coded according to predefined analytical dimensions derived from the study objectives, including organizational design, governance arrangements, management integration, service delivery, and reform outcomes. New codes emerging from the data were also incorporated during the review process.

**(2) Theme development:** Similar codes were grouped into broader thematic categories. Through constant comparison across different data sources, the research team identified four major themes: organizational structure, management pathways, operational mechanisms, and service outcomes.

**(3) Cross-source verification:** Evidence from policy documents, institutional statistics, and interview materials was compared and triangulated to verify consistency and improve the credibility of the findings. Where discrepancies appeared, the research team revisited the original materials and discussed alternative interpretations.

**(4) Team discussion and interpretation:** Three core researchers independently reviewed the coded materials and preliminary themes. Regular meetings were conducted to discuss interpretations, resolve disagreements, and refine the analytical framework until consensus was reached.

**(5) Case synthesis and lesson extraction:** Based on the validated themes, the team developed a comprehensive narrative of the Shaxian CMC reform process and extracted key experiences and policy implications that may be relevant to other settings.

## Construction Measures for the County-level Medical Community (CMC) of Shaxian, Sanming

### Organizational Structure

Shaxian District in Sanming operates under a unified organizational framework, adopting the model of “one leadership team, two signboards, and integrated management”. This approach facilitates the reorganization of the district’s medical and health institutions, forming a cohesive county-level medical community with Shaxian General Hospital at its core. The system is structured as a three-tier healthcare service framework, wherein the county-level public hospital assumes a leadership role, township health centers function as hubs, and village clinics provide foundational support [33]. Furthermore, a highly unified model is implemented across the county, township, and village levels, granting Shaxian General Hospital full autonomy in managing personnel, internal allocation, equipment, drug procurement, operational oversight, and financial administration through a “unified legal entity” structure [34]. This establishes a system marked by unified budgeting and integrated management. The operational mechanism promotes graded diagnosis and treatment, with the principle of “primary diagnosis at grassroots level, two-way referral, differentiated treatment of acute and chronic conditions, and inter-level coordination” steadily advancing.

### Management Pathways

Shaxian District in Sanming has implemented an integrated management model known as the “Eight Unifications”:

**Human Resources** [35]**:** A comprehensive talent recruitment plan is implemented, with an incentive system designed to attract high-quality medical and health professionals back to Sanming. Staffing quotas were expanded, and tailored policies were adopted to address fundamental workforce shortages. Competitive benefits, including preferential housing policies, allowances, and enhanced medical and educational security, are provided to attract talent. Additionally, targeted initiatives are in place to recruit young medical graduates into the grassroots healthcare workforce.

**Medical Services:** Led by Shaxian General Hospital, continuous professional development is promoted through ongoing clinical training aimed at enhancing clinical protocols, treatment techniques, quality of care, and discipline management within the county-level medical community.

**Financial System:** A “unified leadership, centralized management” financial system is adopted, focusing on scientific budgeting, cost accounting, and effective cost control. The system includes standardized procedures for chief accountant duties, budget management, expenditure oversight, and a full-cost accounting framework for hospitals.

**Performance Evaluation** [36]**:** A unified performance evaluation system is implemented across county, township, and village levels, with dynamic adjustments to the evaluation criteria and continuous refinement of how evaluation results are applied. A health-centered compensation system aligns staff remuneration with public health outcomes. Various compensation models, including annual salary schemes for hospital directors, goal-based salary systems, and position-based salary systems, are explored to gradually align employee pay with public health performance.

**Resource Allocation:** Emphasis is placed on balanced resource distribution within the CMC, with a particular focus on decentralizing high-quality resources to grassroots levels. Programs are launched to redistribute medical resources, talent, and disease management efforts to lower levels of the healthcare system.

**Centralized Procurement** [37]: Leveraging the “Sanming purchasing alliance” (a regional volume-based drug and consumables procurement platform pioneered in Sanming to reduce costs through centralized negotiation and bulk purchasing), the internal procurement of drugs and medical consumables within the CMC is centralized. This strengthens catalog management, distribution processes, settlement protocols, and supervision.

**Information Technology Development:** The development of information technology has improved the operational efficiency and quality of county-level medical communities. Sanming City has established a city-wide health information platform, laying the foundation for data interoperability within the county-level medical communities. Based on this, the digital platform can support daily remote collaboration. Sanming City has built six telemedicine centers and established comprehensive channels for clinical, teaching, and research cooperation with top-tier national hospitals. In 2024 alone, more than 34,000 remote consultations were conducted, and the mutual recognition rate of examination results among primary healthcare institutions exceeded 95%. In addition to these fundamental capabilities, the CMC is committed to developing advanced digital applications for comprehensive health management, including using big data to track residents’ health information throughout their entire life cycle, developing health management platforms, and deploying intelligent diagnostic tools in primary healthcare institutions.

**Medical Insurance Prepayment** [38]: A county-level medical insurance fund adopts a package payment system characterized by “total package payment, no reimbursement for overspending, and surplus retention”. A diversified payment system is established, centered on the C-DRG bundle payment (China’s localized version of Diagnosis-Related Groups for bundled payment), integrating multiple payment methods such as project-based payments, per diem (bed-day) payments, and day surgery payments.

### Key Barriers and Solution Pathways

The advancement of the CMC reform was inevitably accompanied by adjustments in interest structures and inherent institutional tensions. Resource integration entails a redistribution of benefits, while relevant administrative functions are dispersed across multiple governmental departments, making it difficult for the health authority alone to coordinate effectively. In response, Sanming established a medical reform leading group headed by senior municipal leaders and delegated authority to the general hospital, with a unified legal representative to ensure a cohesive “one family” decision-making structure.

Once governance authority was clarified, human resources emerged as a prominent challenge in the early stage of reform. Differences in institutional status between county hospitals and township health centers constrained cross-institutional mobility. Sanming addressed this bottleneck by piloting a new staffing model in which the general hospital centrally coordinated staffing quotas across the CMC. Through the principles of “county-managed township employment” and “township-managed village employment,” recruitment and deployment of grassroots personnel were unified and streamlined.

Beyond personnel issues, finance constituted another core barrier. To dismantle the traditional incentive structure linking income to drug sales, consumables, and service volume, Sanming prioritized reform of the compensation system. An annual post-based salary system was adopted for all staff, with unified salary determination, structure, and payment across CMC institutions. This arrangement fostered a more cohesive interest-sharing mechanism across facilities at different levels.

Through coordinated adjustments in governance authority, personnel management, and financial incentives, Sanming established a foundational reform loop centered on “power, personnel, and finance,” laying the institutional groundwork for the subsequent refinement of CMC operational mechanisms.

### Operational Mechanism

Before the reforms, Sanming City in Fujian Province was an economically underdeveloped region facing immense pressure, with its medical insurance fund on the verge of bankruptcy. To fundamentally reverse this situation, Sanming first initiated reforms in the domain of pharmaceuticals. By eliminating profits from drug sales, implementing zero-markup policies, adopting joint price negotiations for medicines and consumables, and strengthening the monitoring of key drugs, the city effectively reduced inflated drug and consumable prices. These measures were complemented by adjustments to service pricing, which helped recalibrate the broader incentive structure. The reform subsequently expanded to the domain of medical services. For medical staff, the reform introduced a position-based annual salary system to strengthen performance incentives. For the county-level medical community as a whole, Sanming adopted a mechanism whereby the medical insurance fund is allocated through a global-budget payment mechanism and any surplus can be retained, thereby aligning compensation and incentive structures. Beyond the bundled payment of the insurance fund, on the medical insurance side, Sanming also integrated the Urban Employee Basic Medical Insurance, Urban Resident Basic Medical Insurance, and the New Rural Cooperative Medical Scheme under unified municipal-level management. With these adjustments, coordination across healthcare delivery, medical insurance, and the pharmaceutical sector was realized in a substantive way.

Importantly, under the unified leadership of the Medical Reform Leading Group, a range of government departments participated in cross-sector collaboration beyond the three core sectors. As noted earlier, the staffing authority played a key role in reforms related to personnel management, while departments such as finance, development and reform, and education provided essential institutional and resource support. Against this backdrop of system-wide coordination, the construction of Sanming’s CMC gradually developed a distinctive operational mechanism.

The operational mechanisms of the Shaxian CMC in Sanming embody a coherent logic that links resource integration, internal governance, and external service provision. First, given the historically limited capacity of primary healthcare institutions in Shaxian, independent operation made it difficult for them to sustain service volume and financial viability [39]. The establishment of a CMC enabled the integration of local health systems and resources, with the general hospital providing survival and development incentives to grassroots institutions through technical support, resource allocation, and referral mechanisms. From the perspective of health system governance theory, the “unified legal entity” helps ensure policy consistency and overall resource optimization within the consortium [40], which is particularly suited to the early stage of consortium development. At the same time, drawing on resource dependence theory, primary institutions gain access to patient flows, financial support, and technical guidance under this arrangement, thereby maintaining basic operations, improving service capacity, and enhancing overall efficiency [41]. Second, to ensure efficient internal functioning, the consortium established a coordinated “Eight Unifications” management system covering human resources, finances, supplies, and other domains. For personnel, unified allocation and two-way mobility ensured sufficient staffing for primary facilities while training and technical support improved service capacity. For supplies, unified drug formularies, procurement of consumables, and equipment management safeguarded quality, safety, and standardization. For finances, a “single-account” system centralized budgeting and allocation, avoiding resource fragmentation and conflicts of interest. From the perspective of integrated care theory, the “Eight Unifications” represent organizational-level integration that advances functional and service integration at the management level, breaking down barriers between departments and tiers, fostering continuity and coordination of care, and enabling smooth patient transfers across facilities. Notably, information technology plays a critical enabling role in this process, with unified digital platforms supporting real-time communication and providing data for managerial oversight [42,43].

Third, once organizational and internal governance structures are consolidated, the consortium must deliver services externally, with the core objective of safeguarding population health. In practice, this involves measures such as medical–prevention integration, the establishment of family physician and health manager systems, and comprehensive chronic disease management. These efforts ensure that healthcare delivery addresses both immediate medical needs and long-term health outcomes.

Finally, with respect to oversight and sustainability, the bundled payment of health insurance funds and retention of surpluses create incentives for financial autonomy at the consortium level. While such mechanisms may carry risks of excessive cost containment or under-provision of necessary services, in Sanming these risks have been offset through performance assessments oriented toward health outcomes and service quality. Specifically, evaluations emphasize not only financial balance but also population health indicators, patient satisfaction, and care quality, ensuring that hospitals pursue efficiency without compromising essential services.

Overall, the Shaxian CMC established an integrated organizational structure covering county, township, and village levels, grounded in unified legal governance and a tiered diagnosis and treatment system. It adopted the “Eight Unifications” to consolidate the management of human resources, finance, medical supplies, and service delivery. Digital infrastructure and information platforms were introduced to enable resource sharing, data interoperability, and coordinated services. Bundled medical insurance payments and performance evaluations further reinforced patient-centered care and health-oriented accountability. This framework has enhanced resource allocation, service continuity, and policy coherence. It provides a valuable example of China’s approach to integrated healthcare reform under resource constraints and offers practical lessons for other developing countries.

Taken together, these mechanisms constitute the analytical logic derived from this case: governance coordination creates institutional authority, the Eight Unifications support organizational integration, and the triple-linkage reform realigns incentives for health-oriented service delivery.

**Figure 1:**
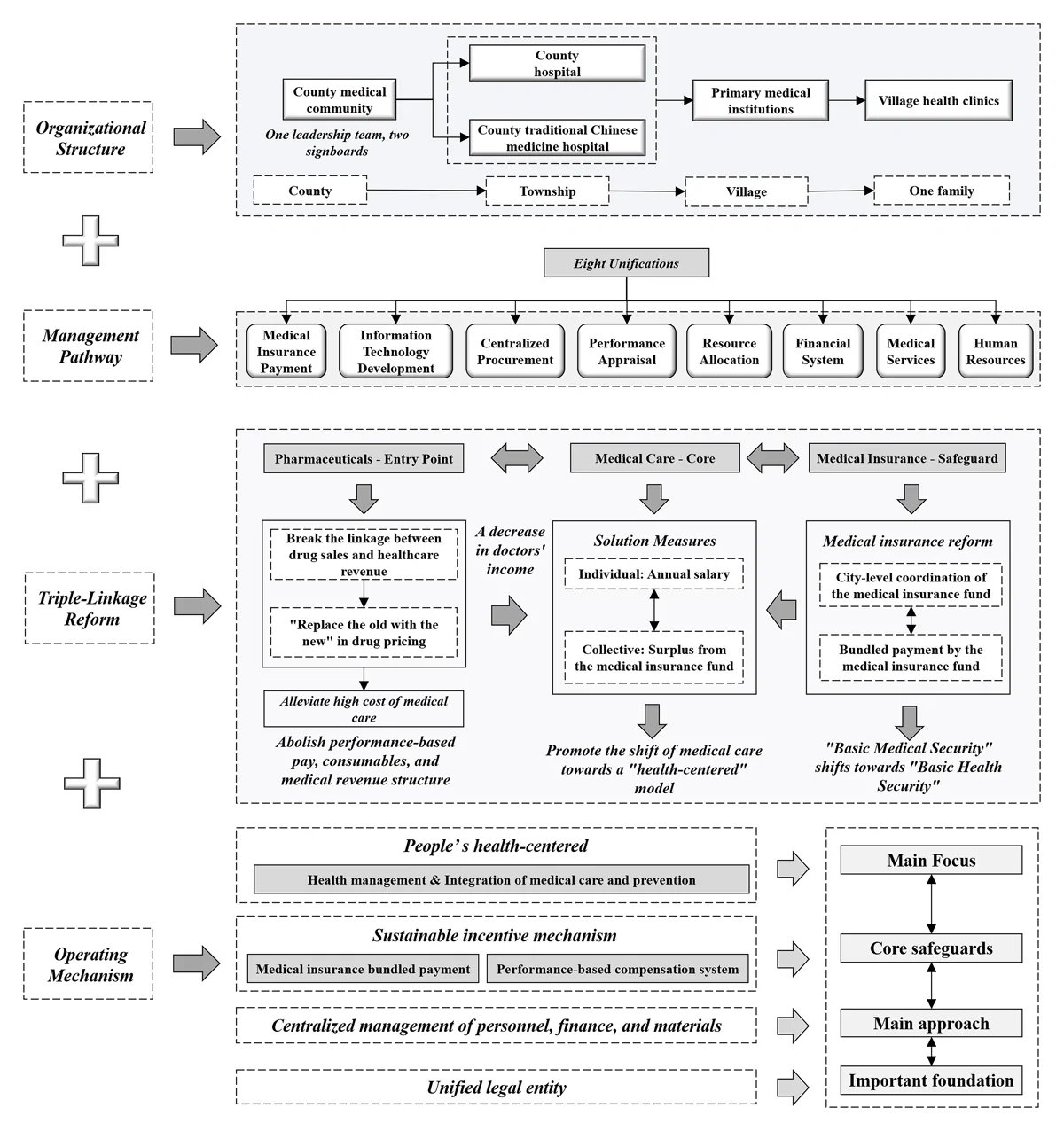
Operational Mechanism for the County-Level Medical Community (CMC) of Shaxian, Sanming.

Figure 1 illustrates how these mechanisms interact within the analytical framework of the Shaxian CMC.

## Contextual Comparison with Selected Integrated Care Arrangements

Because these arrangements operate at different system levels, this section uses selected integrated care arrangements as contextual reference points rather than strictly equivalent comparison units. These include Kaiser Permanente-type integrated delivery systems in the United States, GP-led primary care coordination in the United Kingdom, AIC-led health and social care coordination in Singapore, Japan’s community-based integrated care system, and the Shaxian CMC in China. The comparison focuses on differences in integration logic, governance arrangements, incentive mechanisms, and the role of primary care. Table 2 summarizes these contextual differences.

**Table 2:** Contextual Comparison of Selected Integrated Care Arrangements.


DIMENSIONS	UNITED STATES: KAISER-TYPE INTEGRATED DELIVERY	UNITED KINGDOM: GP-LED PRIMARY CARE	SINGAPORE: AIC-LED COORDINATION	JAPAN: COMMUNITY-BASED INTEGRATED CARE	CHINA: SHAXIAN CMC

Core Driver	Market Competition & Organizational Merger	Tax Funding & Regulation	Corporate Management & Market Competition	Legislation & Policy Mandate	Government-Led Systemic Reform

Key Integration Mechanism	Vertical Integration within a closed system	Strict Tiered Referral System	Horizontal Integration into Corporate Groups	Legally Enforced Medical Circles	Unified Legal Entity & Economic Incentive Realignment

Organizational Structure	Single HMO Entity	Purchaser-Provider Split	Competing Corporate Groups	Legally Defined Private Practices	One Leadership, Two Signboards; Unified County-Town-Village System

Management Focus	Standardized Care Protocols	National Standards & Targets	Corporate Profitability & Efficiency	Professional Autonomy within Rules	The “Eight Unifications”; HR, Finance, Procurement, IT, Performance, etc.

Primary Payment Method	Capitation	Capitation & Budgets	DRG & Global Budgets	Fee-for-Service	C-DRG Bundle Payment; Total Package Payment with Surplus Retention

Pharmaceutical Management	Internal Formulary & Supply Chain	National Price Negotiation	Market-based with Govt Control	Nationally Regulated Pricing	Centralized Volume-Based Procurement

Role of Government	Regulator	Planner, Funder, Regulator	Regulator & Market Architect	Regulator & Legislator	Top-Down Designer & Lead Reformer

Defining Unique Feature	Prepaid, integrated HMO	Universal, tax-funded system	Corporate groups with co-payments	Legally hierarchical private system	Incentive Realignment. Directly tackles the root cause through government-powered systemic overhaul.


The Shaxian case is distinctive in three core aspects:

1. Government as the System Architect: Unlike models that rely on market or administrative mechanisms, the Shaxian reform was driven by a strong, top-down government mandate to rescue a bankrupt insurance fund and combat systemic inefficiency. This aligns with the PCIC framework’s emphasis on strong public leadership and governance to reorient health systems around people’s needs.
2. “Triple-Linkage” as the Core Strategy: It synchronizes reforms in healthcare delivery, medical insurance, and pharmaceutical supply, using centralized drug procurement to generate savings and then strategically reinvesting those savings into revised service prices and physicians’ salaries via C-DRG bundle payment. This multi-level operational integration resonates with the RMIC’s focus on functional, clinical, and professional integration, yet goes further by directly linking financial incentives to performance and outcomes.
3. “Unified legal entity” for holistic control: The “Eight Unifications” under a single legal entity allow for unprecedented control over personnel, finances, and resources, ensuring policy consistency and enabling the effective realignment of incentives across the entire county-level health system. This model of normative and systemic integration offers a contrast to the more decentralized or partnership-based approaches seen in many high-income countries.

The Shaxian model provides a useful reference for other regions, particularly low- and middle-income countries, facing rising healthcare costs and misaligned incentives. However, its strong dependence on centralized governance may limit direct transferability to settings with more fragmented systems or lower administrative capacity. Future comparative studies could examine how specific design features, including incentive-realignment mechanisms, can be adapted to diverse health system contexts.

## Experiences and Insights

### Adapt to Local Conditions and Emphasize Government Responsibility

During his visit to Sanming, General Secretary Xi Jinping emphasized the necessity of tailoring the Sanming model to fit local contexts. The core of this model is its unwavering commitment to the principle of “people first, life first”. This philosophy is deeply rooted in a strong sense of government responsibility. The Sanming reform was consistently driven by top government leaders, supported by strong cross-sectoral coordination mechanisms, which ensured stable political commitment and policy implementation. Such an institutional environment may not be readily present in other countries or regions. In some contexts, limited fiscal capacity of local governments, insufficient interdepartmental collaboration, or stronger autonomy of primary care institutions may constrain the implementation of similar measures. Therefore, when drawing on the Sanming experience, other countries and regions, particularly LMICs, must carefully assess the compatibility of local political, fiscal, and governance conditions. Where such enabling conditions exist, it is crucial to emphasize government responsibility, and governments should enhance their roles to ensure that financial support is timely and that management remains within appropriate boundaries [44].

### Enhance the Capabilities of Leading Hospitals to Ensure Quality

In the construction of the Shaxian CMC, the leading hospital plays a pivotal role as the central pillar of healthcare delivery. Its primary function is to ensure that patients do not need to seek treatment outside the county for serious illnesses. Therefore, it is crucial to continuously improve the comprehensive service capacity of the leading hospital, particularly by focusing on the development of key specialties. This involves a detailed understanding of the local disease profile and the development of targeted healthcare solutions. Furthermore, integrating existing medical resources and fostering the growth of high-potential departments and specialties within the whole county will lead to a differentiated development strategy. In addition to enhancing internal hospital operations, leading hospitals should play a supervisory role in managing grassroots healthcare institutions, setting unified technical standards and treatment protocols, and ensuring internal quality consistency across the medical community. The hospital’s modern management practices must also be elevated by optimizing service workflows, enhancing information technology infrastructure, and ensuring standardized, scientific, and efficient management. Finally, establishing external collaborations, particularly with high-level hospitals from neighboring regions, is essential for building competitive specialties.

### Focus on the Transformation of Grassroots Services and Strengthen the Foundation

Grassroots medical institutions serve as the foundational healthcare providers within the Shaxian CMC, acting as health guardians for local residents. Their primary responsibility is health management. Therefore, when other countries and regions build integrated care systems in the future, it is essential to focus on improving the service quality at grassroots healthcare institutions by adopting a health-centered organizational model. This could involve accelerating the implementation of a “dual-role management” approach, where specialists in disease management and health management work together. Furthermore, a comprehensive health benefit evaluation and oversight mechanism should be established, integrating indicators such as life expectancy and resident health levels into the medical community’s performance evaluation system. The COVID-19 pandemic has emphasized the critical role of grassroots institutions in managing residents’ health services and public health management, particularly in establishing resident health information systems, improving initial diagnosis mechanisms, and enhancing consultation capacity. Additionally, an efficient referral system, including a green channel for referrals to higher-level hospitals within the medical community, must be established.

### Strengthen Supporting Measures and Ensure Comprehensive Coordination

In Sanming’s medical reform, we can find that the development of a county-level medical community is a multifaceted endeavor that requires the coordinated efforts of various stakeholders. On the one hand, the medical, pharmaceutical, and medical insurance sectors must address structural barriers, align policies, and foster large-scale collaboration [45]. On the other hand, societal involvement is also crucial, requiring continuous improvement in supporting measures and coordination across different initiatives. A key challenge in the construction of a medical community is the shortage of medical professionals. Addressing this requires educational institutions to develop targeted training programs for medical students, and staffing departments to optimize the recruitment and retention of medical personnel through effective incentive structures. Housing departments must also provide housing guarantees for medical professionals. Public awareness campaigns are essential to shift the perception that both minor and major illnesses must be treated in large hospitals. The promotion of health management concepts is critical. Therefore, when promoting relevant experiences in the future, it is vital to enhance service quality, improve compensation and benefits for medical staff, and introduce stronger incentives to retain skilled personnel.

## Conclusion

This study reviews and analyzes the practical experience of building the CMC in Shaxian District, Sanming City, Fujian Province. It deconstructs the institutional framework, organizational structure, and operational mechanisms of the reform, and demonstrates its achievements in strengthening healthcare delivery capacity at the county level and safeguarding population health. The findings provide not only a representative case for understanding the development of CMC in China but also valuable insights for other developing countries seeking to advance integrated care services under resource-constrained conditions. The case further suggests that incentive realignment is an important enabling condition for sustainable county-level integration, especially where fragmented financing and weak primary care capacity constrain service coordination.

Nevertheless, some limitations should be acknowledged. First, as a single case study, the generalizability of the findings requires further validation through multi-region and multi-level comparative research. Second, the data are primarily drawn from policy documents, official statistics, and field investigation materials, with limited longitudinal tracking of patient experiences and long-term health outcomes, which may restrict the comprehensiveness of the evaluation. In addition, as far as the construction of the Shaxian case itself is concerned, any large-scale institutional reform will inevitably face certain difficulties. From the patient perspective, although the reform emphasizes health orientation and equity in service provision, sustained monitoring of patient satisfaction and safeguards against potential under-provision of necessary services under cost-containment pressures remain critical. From a policy perspective, given that the Sanming model itself is still in an ongoing process of experimentation and iteration, its institutional design and implementation effects need to be further tested across longer time horizons and wider contexts.

It is also important to recognize potential challenges in applying this model elsewhere. As discussed above, the success of Shaxian’s reform is deeply rooted in specific governance conditions, including strong government leadership, high policy enforcement capacity, and robust intersectoral coordination. In contexts characterized by different institutional, fiscal, resource, or cultural environments, simple transplantation may encounter obstacles such as weak policy execution, uneven resource allocation, or fragmented governance. Even so, several core principles of the Sanming model carry broader relevance: (1) upholding government responsibility and ensuring public financial input to provide institutional guarantees for reform; (2) promoting resource integration and unified governance to enhance county-level efficiency; (3) establishing performance evaluation and incentive mechanisms oriented toward health outcomes and service quality, thereby guiding medical practices toward health-centered goals; and (4) leveraging digital technologies to facilitate resource sharing and service coordination. Thus, the Sanming model should be understood as a reform approach that can be flexibly adapted rather than a rigid “template.” Its effective dissemination requires careful alignment with local governance structures, fiscal and institutional capacities, and sociocultural environments to ensure meaningful and sustainable impact.
